# Supplementary material for: Genetic influences on circulating retinol and its relationship to human health
Source: Nat Commun. 2024 Feb 19;15:1490. doi: 10.1038/s41467-024-45779-x (PMC10876955; doi:10.1038/s41467-024-45779-x)
Supplement: Supplementary file 3 — Description of Additional Supplementary Files [file 41467_2024_45779_MOESM3_ESM.pdf]

Description of additional supplementary files

File Name: Supplementary Data 1-15

Description:

Supplementary Data 1: Estimated mean chi-square values for retinol genome-wide meta-analyses

Supplementary Data 2: SNP heritability estimates

Supplementary Data 3: Replication of lead SNPs in TwinsUK

Supplementary Data 4: Association of circulating retinol lead SNPs with effect sizes from a self-reported retinol intake GWAS (dietary questionnaire derived)

Supplementary Data 5: Non-synonymous suggestively significant retinol associated variants

Supplementary Data 6: Gene-based association using rare variants (Cauchy aggregation)

Supplementary Data 7: Gene prioritisation results (METSIM+INTERVAL)

Supplementary Data 8: Differential expression analysis of FOXP2 overexpression in an oesteosarcoma cell line versus wild type (transcriptome-wide after QC)

Supplementary Data 9: Enriched ontological pathways for genes associated with FOXP2 overexpression (Bonferroni, |log2FC| > 1.5)

Supplementary Data 10: Enriched ontological pathways for genes associated with FOXP2 overexpression (Bonferroni, |log2FC| > 2)

Supplementary Data 11: Enriched ontological pathways for genes associated with FOXP2 overexpression (Bonferroni, |log2FC| > 5)

Supplementary Data 12: Circulating retinol transcriptome-wide association study (TWAS)

Supplementary Data 13: Circulating retinol proteome-wide association study (PWAS)

Supplementary Data 14: Mendelian randomisation using finemapped pQTLs as IVs

Supplementary Data 15: Enriched ontological pathways for prioritised retinol associated genes

File Name: Supplementary Data 16-30

Description:

Supplementary Data 16: Estimated causal effects of retinol across outcomes in IEUGWASdb (RBP4 IV)

Supplementary Data 17: Post-MR colocalisation analyses of a key RBP4 IV derived causal estimates

Supplementary Data 18: Estimated causal effects of retinol across outcomes in IEUGWASdb (All IVs)
Supplementary Data 19: Tiering system applied to causal estimates in IEUGWASdb using all IVs (Tier #2)
Supplementary Data 20: Tiering system applied to causal estimates in IEUGWASdb using all IVs (Tier #2 and Tier #3)
Supplementary Data 21: Causal estimates of body-fat percentage on retinol association brain regions/measures
Supplementary Data 22: Causal estimates on binary outcomes featured in FinnGen release 8 (All MR tests)
Supplementary Data 23: Tier #3 causal estimates, FinnGen release 8
Supplementary Data 24: Effects of traits on retinol as outcome
Supplementary Data 25: Signature matching (individual compounds)
Supplementary Data 26: CMAP-GSEA - pertubagen signatures aggregated to gene-sets
Supplementary Data 27: Reverse MR for Tier #2 and Tier #3 retinol causal estimates
Supplementary Data 28: Reverse causality - FinnGen Tier #3 traits
Supplementary Data 29: Tuning of the retinol PGS in TwinsUK
Supplementary Data 30: Association of retinol PGS with normative deviations for age

File Name: Supplementary Information
Description:
Supplementary Figure 1. Partitioned SNP heritability estimates amongst diverse tissues and cell-types for circulating retinol
Supplementary Figure 2. Empirical Bayes' estimation of non-null effects on vitamin D (25-hydroxyvitamin D_3) genome-wide, stratified by bins of ascendingly sorted LD score based on magnitude
Supplementary Figure 3. Per-cohort effects of the circulating plasma retinol lead SNPs (METSIM+INTERVAL meta-analysis)
Supplementary Figure 4. Genome-wide heterogeneity in the METSIM+INTERVAL meta-analysis (HapMap3 SNPs)
Supplementary Figure 5. The effect of circulating retinol associated lead SNPs on estimated dietary retinol intake

Supplementary Figure 6. Per-cohort effects of circulating retinol lead SNPs as derived from the METSIM+INTERVAL+ATBC+PLCO meta-analysis

Supplementary Figure 7. Correlation between normalised effect sizes of phenome-wide Mendelian randomisation of circulating retinol (MR Z-score) using a single IV in RBP4 versus all independent genome-wide significant SNPs as IVs

Supplementary Figure 8. Exploring the impact of model residual standard error on the IVW-multiplicative random effects (MRE) estimates relative to that of the IVW fixed effects (FE) causal estimates of circulating retinol as the exposure

Supplementary Figure 9. Genetic estimates of the relationship between circulating retinol and binary disease endpoints in the Finnish population

Supplementary Figure 10. Mendelian randomisation causal estimates (IVW-MRE) of circulating retinol on the odds of neoplastic diagnoses derived from cancer registry data collated by FinnGen (release 8)

Supplementary Figure 11. Results from comparing competing models in the CAUSE framework for the effect of serum creatinine on circulating retinol

Supplementary Figure 12. The association between retinol PGS and the odds of an individual falling outside of the normative range of measured circulating retinol for a given age

Supplementary Figure 13. Example of normative model of measured circulating retinol with age

Supplementary Figure 14. Gene-wise plot of the biological coefficient of variation (BCV) versus transcript abundance log counts per million (CPM) from RNAseq data derived from an osteosarcoma cell line

Supplementary Figure 15. GAMLSS model fits for the effect of age on circulating retinol in the entire TwinsUK cohort

Supplementary Figure 16. GAMLSS model fits for the effect of age on circulating retinol in the half of TwinsUK cohort (unrelated individuals)

Supplementary Note 1: Gene prioritisation within the circulating retinol genome-wide significant loci

Supplementary Note 2: Comparing the inverse variance weighted estimator with multiplicative random effects versus fixed effects in the absence of instrumental variable heterogeneity

Supplementary Note 3 Multivariable Mendelian randomisation models testing the effects of serum creatinine and major lipid species on circulating retinol

Supplementary Note 4: Normative modelling

Supplementary Note 5: Profiling of plasma retinol in the INTERVAL cohort
