# Supplementary material for: Genetic influences on circulating retinol and its relationship to human health
Source: Nat Commun. 2024 Feb 19;15:1490. doi: 10.1038/s41467-024-45779-x (PMC10876955; doi:10.1038/s41467-024-45779-x)
Supplement: Supplementary file 5 — Reporting Summary [file 41467_2024_45779_MOESM5_ESM.pdf]

nature portfolio

Corresponding author(s): William Reay, Murray Cairns

Last updated by author(s): 01/06/2024

Reporting Summary

Nature Portfolio wishes to improve the reproducibility of the work that we publish. This form provides structure for consistency and transparency in reporting. For further information on Nature Portfolio policies, see our Editorial Policies and the Editorial Policy Checklist.

Statistics

For all statistical analyses, confirm that the following items are present in the figure legend, table legend, main text, or Methods section.

n/a	Confirmed	
☐	☒	The exact sample size (*n*) for each experimental group/condition, given as a discrete number and unit of measurement
☐	☒	A statement on whether measurements were taken from distinct samples or whether the same sample was measured repeatedly
☐	☒	The statistical test(s) used AND whether they are one- or two-sided<br>*Only common tests should be described solely by name; describe more complex techniques in the Methods section.*
☐	☒	A description of all covariates tested
☐	☒	A description of any assumptions or corrections, such as tests of normality and adjustment for multiple comparisons
☐	☒	A full description of the statistical parameters including central tendency (e.g. means) or other basic estimates (e.g. regression coefficient) AND variation (e.g. standard deviation) or associated estimates of uncertainty (e.g. confidence intervals)
☐	☒	For null hypothesis testing, the test statistic (e.g. *F*, *t*, *r*) with confidence intervals, effect sizes, degrees of freedom and *P* value noted<br>*Give P values as exact values whenever suitable.*
☐	☒	For Bayesian analysis, information on the choice of priors and Markov chain Monte Carlo settings
☒	☐	For hierarchical and complex designs, identification of the appropriate level for tests and full reporting of outcomes
☐	☒	Estimates of effect sizes (e.g. Cohen's *d*, Pearson's *r*), indicating how they were calculated

Our web collection on statistics for biologists contains articles on many of the points above.

Software and code

Policy information about availability of computer code

Data collection	No custom software was used for data collection in this study
Data analysis	All code used in this study has been publicly deposited on GitHub - https://github.com/Williamreay/Retinol_GWAS_code<br>The software used was a follows:<br>FIZI v0.7.2<br>PLINK v2.00a3LM AVX2 Intel<br>METAL (version March 2011)<br>FUMA v1.4.1<br>Cauchy (ACAT) code adapted from https://github.com/yaowuliu/ACAT.<br>ldsc<br>LDAK v.5.2<br>ashR R package v2.2-54<br>edgeR package version 3.34.0<br>g:Profiler version e109_eg56_p17_1d3191d<br>FUSION vNov16, 2021<br>TwoSampleMR package v0.5.6,<br>IEUGWASdb v6.9.2<br>coloc v5.1.0<br>CAUSE R package v1.2.0<br>MendelianRandomization v0.6.0<br>gamlss R package v5.4.12

The primary analyses in this manuscript were performed either on a MacBook Pro (OS X: Ventura 13.3), an in-house linux cluster (Ubuntu 18.04.5 LTS), or the High-Performance Computing Research Compute Grid of the University of Newcastle [Red Hat Enterprise Linux release 8.1 (Ootpa)]. The primary R version utilised was version 4.1.1 (2021-08-10), with some additional analyses using R version 4.0.3 (2020-10-10) (linux cluster). The Python version utilised was either Python 2.7.17 or Python 3.6.9, depending on the requirements of the analyses.

For manuscripts utilizing custom algorithms or software that are central to the research but not yet described in published literature, software must be made available to editors and reviewers. We strongly encourage code deposition in a community repository (e.g. GitHub). See the Nature Portfolio guidelines for submitting code & software for further information.

Data

Policy information about availability of data

All manuscripts must include a data availability statement. This statement should provide the following information, where applicable:

- Accession codes, unique identifiers, or web links for publicly available datasets
- A description of any restrictions on data availability
- For clinical datasets or third party data, please ensure that the statement adheres to our policy

The full retinol GWAS summary statistics generated in this study are available at https://doi.org/10.5281/zenodo.7905523. The RNAseq data analysed by this study are available in the Gene Expression Omnibus (GEO) under accession code GSE138938 https://www.ncbi.nlm.nih.gov/geo/query/acc.cgi?acc=GSE138938. The 1000 Genome Project data utilized for LD calculation can be available at ISGR (https://www.internationalgenome.org/data). The datasets used annotation of variants as eQTLs and pQTLs are publicly available from GTEx (https://www.gtexportal.org/home/downloads) and the Chatterjee lab repository (https://nilanjanchatterjeelab.org/pwas/), respectively. Weights used for the estimation of TWAS test statistics are available from the Gusev lab repository (http://gusevlab.org/projects/fusion/). FinnGen GWAS summary statistics utilised in this study are publicly available from the FinnGen website (https://www.finngen.fi/en/access_results). GWAS data from IEUGWASdb utilised in the study for the MR-pheWAS can be publicly accessed from their website (https://gwas.mrcieu.ac.uk/). LD tagging files for the UKBB utilised for heritability estimation can be sourced from the LDAK website (https://dougspeed.com/ldak/). Metabolite raw relative abundances are available for INTERVAL, a cohort included in the GWAS meta-analysis, at https://www.ebi.ac.uk/metabolights/ (project codes: MTBLS833 and MTBLS834).
The TwinsUK data used in this study are available under restricted access to protect participant privacy as outlined by the study protocol of TwinsUK, access can be obtained by approved, bona fide researchers by following the steps detailed by the TwinsUK website: https://twinsuk.ac.uk/resources-for-researchers/our-data/). Researchers wishing to access TwinsUK data must read the data access policy (https://twinsuk.ac.uk/wp-content/uploads/2022/12/DTR_DataAccessPolicy_2022V1.pdf) and complete a “Data Access Proposal Form” for consideration by the TwinsUK Research Executive Committee. Specific enquires related to data access can be directed to victoria.vazquez@kcl.ac.uk .

Research involving human participants, their data, or biological material

Policy information about studies with human participants or human data. See also policy information about sex, gender (identity/presentation), and sexual orientation and race, ethnicity and racism.

Reporting on sex and gender	Sex was included as a covariate in the GWAS analyses, however, no analyses were stratified by sex. Sex was based in those studies on self-reported data.
Reporting on race, ethnicity, or other socially relevant groupings	We defined genetic ancestry based on SNP derived principal component analyses - however, these do not capture the complex sociocultural factors related to race or ethnicity and as such, should be interpreted as a statistical tool only in the context of GWAS.
Population characteristics	Primary GWAS cohorts (drawn from previous studies):METSIM: all participants male, full METSIM genetic cohort reported by Yin et al - aged 45–74 (median=58). ATBC: all male, median age 58 (range 54–62), PLCO: all male, 6median age 5 (61–68). For TwinsUK: (raw data analysed in this study) The mean age of participants at each timepoint was 51.5 (SD = 8.41), 58.6 (SD = 8.38), and 64.7 (SD = 8.41), respectively. There were only a very small number of males in this cohort (~ 3%), so only females were retained for further analysis (N = 1696) due to this imbalance in sex composition.
Recruitment	All the primary GWAS studies were directly recruited, and as such, may be subject to selection bias.
Ethics oversight	The TwinsUK Resource Executive Committee (project ID: E1205) approved the use of the TwinsUK dataset. All remaining analyses used publicly available data or data accessed through private communication. NCI approved the final methods and results of the manuscript.

Note that full information on the approval of the study protocol must also be provided in the manuscript.

Field-specific reporting

Please select the one below that is the best fit for your research. If you are not sure, read the appropriate sections before making your selection.

☒ Life sciences ☐ Behavioural & social sciences ☐ Ecological, evolutionary & environmental sciences

For a reference copy of the document with all sections, see nature.com/documents/nr-reporting-summary-flat.pdf

Life sciences study design

All studies must disclose on these points even when the disclosure is negative.

Sample size	We used all available data for the retinol GWAS, and as such, there was no predetermined sample size.
Data exclusions	Variants were excluded from the meta-analysis if they were not in all cohorts considered. In regards to TwinsUK, Relatedness testing in both sub-cohorts containing one of the two possible twin pairs was performed separately using KING as implemented by plink2 (PLINK v2.00a3LM AVX2 Intel), with one participant from third-degree relative or greater pairs randomly removed.
Replication	Replication was performed in TwinsUK - After merging with the genotyped split twin cohorts, as described above, there were up to 916 and 717 genotyped participants with measured retinol at three timepoints in each subset, respectively. Six GWAS were performed: in each sub-cohort (one or two), multiple linear regression was utilised to test the additive effect of each variant on measured retinol at one of the three measured time-points covaried for age, five SNP derived PCs, and metabolomics batch. These GWAS was performed using the --glm flag in plink2, resulting a 9,051,192 by three matrix of estimated retinol effect sizes for both sub-cohorts.<br>Considering the mean association across all timepoints retinol was measured, as well as the twin pairs separately, 7 out of the 8 lead SNPs in the loci had effect sizes in the same direction, which was greater than expected by chance alone (Binomial P = 0.035, Supplementary Data 3). Of these, the TTR, GCKR, FOXP2, and PPP1R3B lead SNPs, denoted as such based on their closest TSS, were at least nominally significant at one or more of the measured timepoints.
Randomization	Not applicable as an observational study which was performed on a quantitative trait (measured retinol).
Blinding	Not applicable as an observational study which was performed on a quantitative trait (measured retinol), therefore was no requirement for blinding to perform this study.

Reporting for specific materials, systems and methods

We require information from authors about some types of materials, experimental systems and methods used in many studies. Here, indicate whether each material, system or method listed is relevant to your study. If you are not sure if a list item applies to your research, read the appropriate section before selecting a response.

Materials & experimental systems

n/a	Involved in the study
☒	☐ Antibodies
☒	☐ Eukaryotic cell lines
☒	☐ Palaeontology and archaeology
☒	☐ Animals and other organisms
☒	☐ Clinical data
☒	☐ Dual use research of concern
☒	☐ Plants

Methods

n/a	Involved in the study
☒	☐ ChIP-seq
☒	☐ Flow cytometry
☒	☐ MRI-based neuroimaging
